# The journey of preproteins across the chloroplast membrane systems

**DOI:** 10.3389/fphys.2023.1213866

**Published:** 2023-06-01

**Authors:** Gent Ballabani, Maryam Forough, Felix Kessler, Venkatasalam Shanmugabalaji

**Affiliations:** Laboratory of Plant Physiology, Institute of Biology, University of Neuchâtel, Neuchâtel, Switzerland

**Keywords:** chloroplasts, preprotein, transit peptides, TOC-TIC, thylakoid

## Abstract

The photosynthetic capacity of chloroplasts is vital for autotrophic growth in algae and plants. The origin of the chloroplast has been explained by the endosymbiotic theory that proposes the engulfment of a cyanobacterium by an ancestral eukaryotic cell followed by the transfer of many cyanobacterial genes to the host nucleus. As a result of the gene transfer, the now nuclear-encoded proteins acquired chloroplast targeting peptides (known as transit peptides; transit peptide) and are translated as preproteins in the cytosol. Transit peptides contain specific motifs and domains initially recognized by cytosolic factors followed by the chloroplast import components at the outer and inner envelope of the chloroplast membrane. Once the preprotein emerges on the stromal side of the chloroplast protein import machinery, the transit peptide is cleaved by stromal processing peptidase. In the case of thylakoid-localized proteins, cleavage of the transit peptides may expose a second targeting signal guiding the protein to the thylakoid lumen or allow insertion into the thylakoid membrane by internal sequence information. This review summarizes the common features of targeting sequences and describes their role in routing preproteins to and across the chloroplast envelope as well as the thylakoid membrane and lumen.

## Introduction

The chloroplast is a member of the plastid organelle family known mostly for its photosynthetic activity though it does perform a vast array of other metabolic activities essential to plant survival, development, and stress responses (Jarvis and López-Juez, 2013). Plastids are the result of an endosymbiotic process that started over a billion years ago (Zimorski et al., 2014). Since that time, most plastid genes have either been lost or transferred to the nucleus (Timmis et al., 2004). Of the 2,000 plus chloroplast proteins only about 10% remain encoded by the chloroplast genome. The nuclear-encoded chloroplast preproteins contain an N-terminal transit peptide (TP). The TP can be compared to a molecular zip code of preproteins to be targeted to the chloroplast and imported via the chloroplast protein import machinery (Lee and Hwang, 2021).

The import mechanism involves multiple steps at different (sub-)organellar locations. Initially, the preprotein is guided through the cytosol accompanied by a chaperone complex until it is handed off at the outer envelope of the chloroplast where the transit peptide makes first contact with the TOC (Translocon at the Outer envelope of the Chloroplast) complex (Flores-Pérez and Jarvis, 2013). This involves the action of the two GTP-binding receptors TOC159 and TOC34. In a process that requires GTP and low concentrations of ATP (0.1 mM), the preprotein is inserted across the large hybrid outer membrane protein-conducting channel that consists of the C-terminal ß-barrel membrane (M-) domain of TOC159 and that of TOC75 (Richardson et al., 2014; Schnell, 2019). At this stage already, the transit peptide is in contact with the intermembrane POTRA-domains of TOC75 and initiates contact with components of the TIC (translocon at the Inner envelope of the chloroplast) complex, namely, TIC22 and TIC20, however, without traversing the inner membrane (Kouranov et al., 1998). In the presence of high concentrations of ATP (> 1 mM) the preprotein crosses the TIC20 inner membrane protein-conducting channel and enters the chloroplast stroma assisted by ATP-dependent motor components (Richardson et al., 2018).

Once inside the stroma, the transit peptide is cleaved by the Stromal Processing Peptidase (SPP) (Richter and Lamppa, 1998). Many imported, mature proteins remain in the stroma and are folded with the help of chaperones. Some proteins, however, are targeted further to the thylakoid membrane or lumen. Thylakoid lumen targeted proteins possess bipartite targeting sequences consisting of a transit peptide followed by a thylakoid targeting signal that engages one of two pathways leading to the thylakoid lumen: The ΔpH-dependent TAT (twin arginine targeting) and SEC (secretory) pathways. However, the Signal Recognition Particle (SRP) pathway inserting proteins into the thylakoid membrane relies on targeting information residing within the mature sequence. Each of these pathways relies on a distinct set of protein components. Thylakoid targeting signals of preproteins are removed by a thylakoid processing peptidase (TPP) which promotes final assembly of the mature proteins leading to functional chloroplasts (Mori and Cline, 2001; Albiniak et al., 2012; Teixeira and Glaser, 2013).

## Protein translocation into the chloroplast

### The primary structures of transit peptides are highly diverse

“Signal Peptide” refers to an endoplasmic reticulum targeting sequence, “pre-sequence” to a mitochondrial one, and “transit peptide” is specific for chloroplast-targeted proteins (Bruce, 2000). In the late 1970s, after the signal hypothesis had been proposed, a study showed that *in vitro* translated Rubisco small unit (RbcS) protein had a higher molecular mass than mature RbcS in plant extracts. It was therefore considered a putative precursor (Dobberstein et al., 1977). The RbcS cDNA was cloned and revealed an N-terminal extension that was not present in the mature RbcS. It was identified as the chloroplast targeting sequence and coined “transit peptide” (Broglie et al., 1981; Coruzzi et al., 1983). Later studies demonstrated that the putative precursor of RbcS was transported into isolated chloroplasts and processed to its mature form (Highfield and Ellis, 1976; Chua and Schmidt, 1978). It has been proposed that transit peptides evolved from antimicrobial amphipathic peptides derived from host cells during endosymbiotic events, an intriguing hypothesis that is supported by experimental evidence (Caspari et al., 2023).

A motif study has shown that transit peptides contain three regions, a N-terminal region lacking charged amino acids, a central one containing hydroxylated amino acids and C-terminal one containing an arginine rich motif. This domain structure may be common to most preproteins (Karlin-Neumann and Tobin, 1986; von Heijne et al., 1989; Bruce, 2001). A later study, reporting extensive mutagenesis of the RbcS transit peptide, provided clues to the existence of FP/RK and MLM motifs in the transit peptide and their vital role in chloroplast protein import (Lee et al., 2006). Site-specific cross-linking experiments with the RbcS transit peptide, demonstrated that the FP/RK motif is important for interaction not only with components of the TOC complex, but also with the TIC20 component of the TIC complex (Richardson et al., 2018). In addition, FGLK is a transit peptide motif that has been characterized as being recurrent in transit peptides and playing an important role in the preprotein recognition by TOC34. The deletion of the FGLK sequence by mutagenesis prevented the preprotein from being translocated into the chloroplast (Chotewutmontri et al., 2012; Holbrook et al., 2016).

Based on a synthetic transit peptide, a study demonstrated that FGLK and FP/RK motifs are essential for RbcS transit peptide function and preprotein targeting of the chloroplast (Lee et al., 2015). Moderate hydrophobicity at the N-terminal region of the transit peptide is important for preprotein recognition, (Bhushan et al., 2006; Lee et al., 2006; Lee et al., 2008). Exchange of basic amino acids (N-terminal region) to acidic amino acids negatively affected preprotein import into chloroplasts (Razzak et al., 2017; Lee and Hwang, 2019). Twin-positive (positively charged amino acids) motifs in the TP appear to play a key role in preprotein import into old *versus* young chloroplasts (Teng et al., 2012). In addition, large scale *in silico* analysis and experimental evidence revealed that the twin-positive motif is important for preprotein import into leucoplasts (Chu et al., 2020).

The importance of proline residues in transit peptides has been demonstrated by comparing the import of preproteins containing proline-rich transit peptides with those lacking proline residues. The mutation of transit peptides by the replacement of prolines by alanines resulted in reduced efficiency of translocation into the chloroplast, specifically concerning transmembrane proteins and proteins prone to aggregation (Lee et al., 2018; Jeong et al., 2021). Proline is an amino acid that tends to disrupt the secondary structures of polypeptides (Guzzo, 1965). As preproteins are believed to be translocated across the TOC complex an unstructured transit peptide as described by the “perfect random coil hypothesis” may be advantageous to initiate the early stages of protein import (von Heijne and Nishikawa, 1991).

### Energetics of translocation across at the chloroplast envelope membranes

The energy requirement of preprotein transport across the chloroplast envelopes was first analyzed in an *in vitro* import assay using isolated chloroplasts that were either light- or dark-adapted. The study showed that import into dark-adapted chloroplasts was compromised (Grossman et al., 1980). Exogenously added ATP rescued imports into dark-adapted chloroplasts, demonstrating that ATP was the primary energy source (Cline et al., 1985). Later studies demonstrated that import of preproteins into chloroplasts was driven by the hydrolysis of ATP inside the chloroplast (Flügge and Hinz, 1986; Pain and Blobel, 1987; Theg et al., 1989). It was then revealed that distinct concentrations of ATP in different compartments defined separate steps of chloroplast protein import. Low concentrations of ATP (50–100 µM) were sufficient for preprotein binding to the surface of the chloroplast, whereas high concentrations of ATP (1 mM or more) were required for protein translocation across the chloroplast envelope (Olsen et al., 1989). The RbcS preprotein could be chemically crosslinked to chloroplast envelope component in an ATP-dependent manner (Perry and Keegstra, 1994).

The energetics findings were exploited to generate preprotein translocation intermediates and isolate the first components of the protein import machinery from isolated pea chloroplasts (Schnell and Blobel, 1993). In these experiments, recombinant preprotein of RbcS fused to two IgG-binding domains of *Staphylococcus aureus* ProteinA (resulting in pS-ProtA) was used as a tool. When incubated at low ATP concentrations, pS-ProtA is stably bound to isolated chloroplasts. pS-ProtA remained sensitive to exogenous protease and the transit peptide was not cleaved. This state defines the “early translocation intermediate”. When incubated at high ATP concentrations, pS-ProtA was fully imported. However, its import could be arrested by chilling on ice. At this stage, pS-ProtA was both accessible to exogenous protease and the transit peptide partially cleaved resulting in mature S-ProtA. Thus, pS-ProtA and S-ProtA had traversed and were now spanning both the outer and inner envelope membrane. This state defines the “late translocation intermediate”. It is important to note that the formation of both the “early” and “late” translocation intermediates critically depended on the presence of the transit peptide in pS-ProtA (Schnell and Blobel, 1993). The production of “early” and “late” translocation intermediates was upscaled from analytical to biochemical quantities allowing their isolation by IgG-affinity chromatography. The “early” translocation intermediate pS-ProtA was associated with three visible bands on a SDS-PAGE gel. These first three proteins were molecularly cloned and sequenced and are now known as TOC159, TOC75 and TOC34 (Kessler et al., 1994; Schnell et al., 1994). The three form the core of the TOC-complex as it is widely accepted today. In addition to the three core components of the TOC-complex, the “late” translocation intermediate pS-ProtA and S-ProtA associated with two more bands. One is known today as TIC110 while the second one, named IAP36 at the time, was never identified (Schnell et al., 1994). To this day, the role of TIC110 in chloroplast protein import remains contested and is notably absent from algal protein import complexes (Ramundo et al., 2020).

### Translocon complexes at the inner and outer chloroplast membranes

**FIGURE 1:**
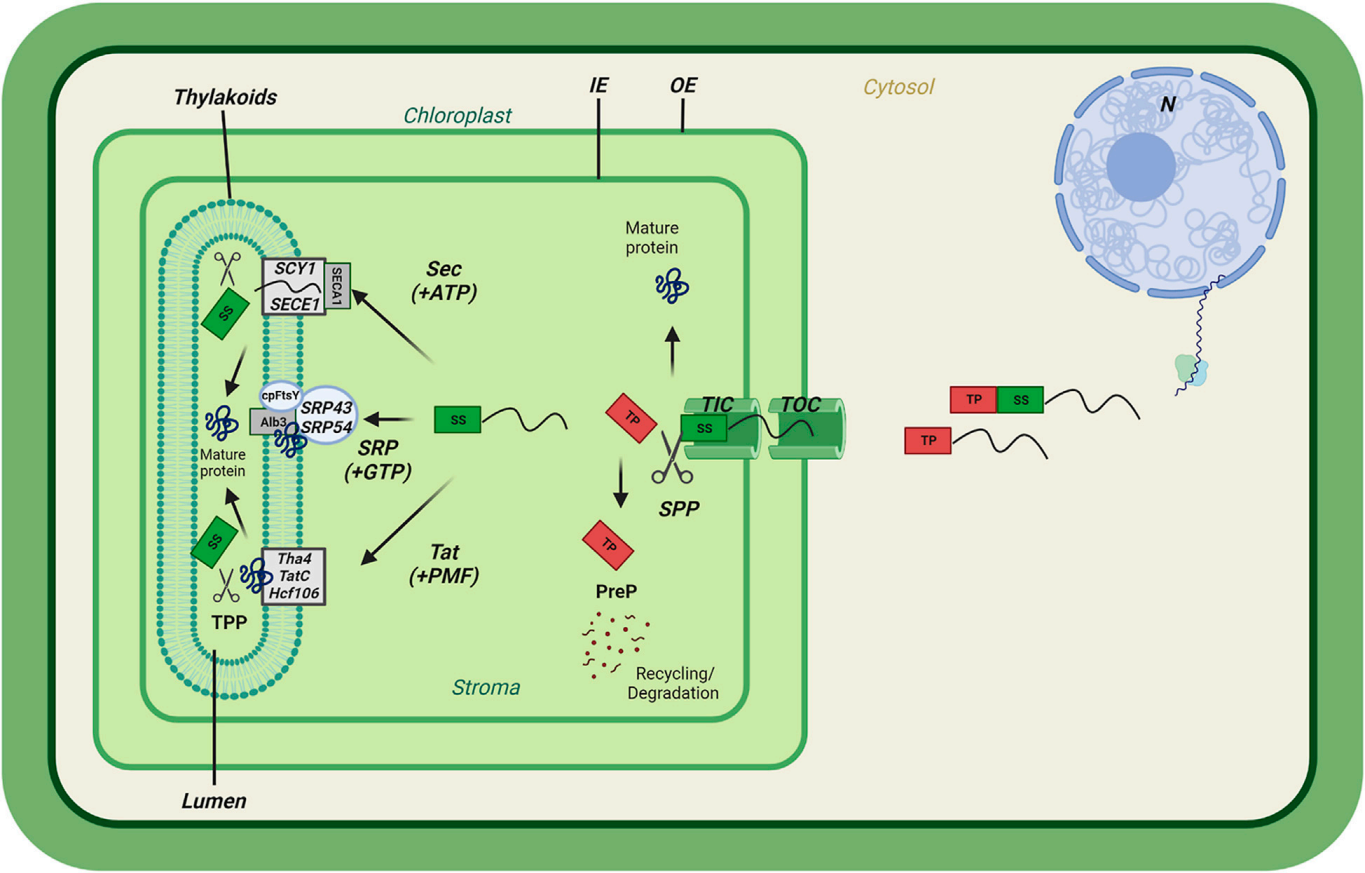
Preproteins translocation into chloroplast membrane systems. General scheme of chloroplast import of nuclear-encoded preproteins containing a transit peptide (TP) followed by a thylakoid Signal sequence (SS). Preprotein translocation passes through the Translocons at the Outer envelope (OE) of the Chloroplast (TOC) and the Inner envelope (IE) of the chloroplast (TIC). Upon entry, the transit peptide is cleaved by the Stromal Processing Peptidase (SPP) and processed by the PreP protease for recycling/degradation. The thylakoid-targeted proteins pass through either the Twin Arginine Transport (Tat) pathway requiring the Proton Motive Force (PMF), the Sec requiring ATP, or the Signal Recognition Particle (SRP) pathway. Upon thylakoid membrane insertion, the thylakoid signal sequence (SS) is cleaved by the Thylakoid Processing Peptidase (TPP), completing the final import step.

The large majority of chloroplast proteins are imported via the TOC-TIC complexes (Figure 1). The first components were identified in the beginning of 1990s as a result of studies on isolated pea chloroplasts and revealed three components of the outer and one at the inner envelope membranes (namely, Import intermediate Associated Proteins or Outer Envelope Protein), IAP/OEP34, IAP/OEP75, IAP/OEP86 and Inner Envelope Protein IAP100/IEP110 (Hirsch et al., 1994; Kessler et al., 1994; Perry and Keegstra, 1994; Schnell et al., 1994). These translocon components were renamed according to the TOC–TIC nomenclature as Toc34, Toc75, and Toc159 (for IAP/OEP86) and Tic110 (Schnell et al., 1997). The initial characterization revealed that TOC159 and TOC34 were homologous GTP-binding proteins exposed at the chloroplast surface. They were both sensitive to the addition of exogenous thermolysin protease fulfilling an important criterium for preprotein receptors at the chloroplast surface (Kessler et al., 1994). TOC75 was insensitive to exogenous thermolysin, bore homology to cyanobacterial ß-barrel solute channels related to the β-barrel assembly machinery A (BamA) family fulfilling criteria for a protein-conducting channel at the outer chloroplast membrane. TIC110 had two N-terminal alpha-helices and a large stromal domain suggesting that it may function as scaffold coordinating late translocation functions such as recruitment of chaperones for protein folding and assembly (Kessler and Blobel, 1996).

The presence of GTP-binding proteins in the TOC complex encouraged further energetics experimentation. Preprotein binding to chloroplasts does not only require low concentrations of ATP but also implicates GTP as non- and slowly-hydrolyzable GTP analogs inhibited import. These findings supported the importance of the role of TOC GTPase receptors (Olsen and Keegstra, 1992; Kessler et al., 1994). Apart from the irreversible energy-dependent interactions, the transit peptide is also reversibly bound to TOC159 and TOC75 in an energy-independent way as demonstrated by chemical cross-linking (Ma et al., 1996).

In Arabidopsis as well as other species, both of the GTP-binding TOCs are encoded by multigene families and consequently several isoforms of each have been discovered. The structure of TOC34 as well as those of its homologs consists of two main features, a N-terminal GTPase domain and a single C-terminal alpha-helical membrane-spanning domain followed by a short hydrophilic tail (Jarvis et al., 1998). TOC159 and its three homologs in Arabidopsis (atTOC120, −132, −90) possess a central GTPase (G-) domain, a C-terminal membrane-anchoring (M-) domain, and a N-terminal acidic (A-) domain at the N-terminus (Kubis et al., 2004). The M-domain has now been shown to take on a ß-barrel structure and associate with TOC75 to form a large hybrid channel at the outer chloroplast membrane (Jin et al., 2022; Liu et al., 2023). The A-domains in the four Arabidopsis isoforms of TOC159 are much more divergent than the G- and M-domains and appear to play a role in pre-protein specificity (Agne et al., 2010). It, however, is not clear how the various A-domains distinguish the transit peptides of different classes of preproteins (i.e., photosynthesis-associated *versus* house-keeping) (Bauer et al., 2000; Ivanova et al., 2004). TOC75 belongs to the BamA family with homologs in Gram-negative bacteria as well as mitochondria and plastids (Schleiff and Becker, 2011). Based on these similarities, TOC75 was proposed to function as the protein-conducting channel at the outer membrane of the chloroplast. TOC75 is encoded by a single orthologous gene in the genomes of all plant species sequenced so far. In addition to forming a ß-barrel channel, TOC75 has three N-terminal POTRA (polypeptide transport-associated) domains (Sánchez-Pulido et al., 2003; Srinivasan et al., 2023). The POTRA domain contributes to preprotein recognition and has chaperone-like activity to guide the incoming preprotein across the intermembrane space (Kouranov and Schnell, 1997; Paila et al., 2016; O'Neil et al., 2017).

At the inner envelope membrane, at least two models have been proposed for the TIC complex, the first consisting of the TIC20 (channel) TIC214 (plastid-encoded), TIC100, TIC56, TIC21 and TIC12 forming a 1 MDa complex (Kikuchi et al., 2013) the second consisting of TIC110 and TIC40. Currently, it is not clear whether the second complex functions together with or independently from the 1 MDa TIC complex in land plants. Cryo-EM structures of the Chlamydomonas TOC-TIC holocomplexes, however, did not contain homologs of TIC40 or TIC110 (Jin et al., 2022; Liu et al., 2023). In addition to the aforementioned components the intermembrane space component TIC236 constitutes a physical link between the TOC and TIC complexes (Chen et al., 2018). TIC22, another intermembrane space component, has been proposed to promote preprotein import across both envelope membranes and the intermembrane space besides its function as a chaperone (Kouranov et al., 1999). As preprotein import requires ATP, the existence of ATP-dependent motors has been proposed. However, the exact nature of such stromal import motor(s) is currently contested. On the one hand biochemical and genetic information provide support for a chaperone network consisting of cpHsp70, Hsp90C, and Hsp93) consuming the ATP and energizing translocation (Su and Li, 2010; Inoue et al., 2013; Huang et al., 2016). On the other hand, an alternative stromal motor has been proposed that consists of a 2-MDa ycf2/FtsH1 complex that also has predicted ATP hydrolysis activity (Kikuchi et al., 2018). However, the respective significance of the two proposed motor systems has not been evaluated so far, and neither of the two systems were observed in the currently available Cryo-EM structures (Jin et al., 2022; Liu et al., 2023).

### Transit peptides are cleaved by stromal processing peptidase

Upon entry into the chloroplast stroma and possibly before complete translocation, the transit peptide is cleaved by Stromal Processing Peptidase (SPP) (Figure 1) (Richter and Lamppa, 2002; Richter and Lamppa, 2003). SPP is an M16 metallopeptidase carrying out a function comparable to that of the Mitochondrial Processing Peptidase (MPP), a metalloprotease, involved in the maturation of nuclear encoded proteins imported into mitochondria (Pollock et al., 1988; Braun et al., 1992). SPP, cleaves at a semiconserved motif ((I/V)-X-(A/C)-↓-A (arrow marks cleavage site) at the C-terminus of the transit peptide (von Heijne et al., 1989). Thereby initiating the final steps of preprotein maturation. After transit peptide cleavage, these may include folding and/or assembly in the stroma or insertion into or translocation across the thylakoid membrane. SPP is an essential component of the import mechanism as demonstrated by the aborted seed phenotype observed in the *spp* homozygous knockout mutants (Trösch and Jarvis, 2011). Once cleaved, transit peptides are further degraded by presequence peptidases (PrePs) (Figure 1) (Ståhl et al., 2005).

### Thylakoid membrane targeting sequences and alternative insertion pathway

The thylakoid membrane is home to the light reactions of photosynthesis. For thylakoid biogenesis, assembly of thylakoid luminal and integral membrane proteins is essential. For a considerable number of proteins, the journey therefore is not finished upon arrival inside the chloroplast. Cleavage of the transit peptide may expose a secondary targeting sequence that will engage one of at least two entry pathways to the thylakoids. Two routes exist for entering the thylakoid lumen: the twin-arginine translocase (TAT) that may accommodate folded proteins, or the SEC translocase for unfolded proteins (Figure 1) (Yuan et al., 1994; Mori et al., 1999). In addition, integral thylakoid membrane proteins require the signal recognition particle (SRP) pathway for alternative insertion (Figure 1) (Schünemann, 2007). Interestingly, all three pathways have been conserved from the cyanobacterial ancestor and exist in bacteria and, in the case of the SEC and SRP pathways, in animals to this day.

### SEC translocation mechanism

The SEC pathway is well-known for its evolutionary conserved mechanism (Dalbey and Chen, 2004). In thylakoid targeting signals, the SEC-specific signal sequence has been described as containing three domains, a charged domain at the N-terminal part, a hydrophobic mid-section and C-terminal cleavage domain containing an A-x-A motif set for interaction with the thylakoid processing peptidase (TPP) (Hsu et al., 2011; Celedon and Cline, 2013). SEC1 is the SEC translocase at the thylakoid membrane (Fernandez, 2018). The SEC1 complex contains SCY1 and SECE1 thylakoid membrane protein channels associated with the stromal motor protein SECA1 (Nakai et al., 1994). Nuclear-encoded lumenal proteins are translocated in an unfolded form across the SEC translocase. The N-terminal part of the signal peptide interacts with SECA1 translocation motor and its ATPase activity provides the energy for translocation across the SCY1/SECE1 channel. Subsequently, the signal sequence is cleaved in the thylakoid lumen (Figure 1) (Albiniak et al., 2012). HSP90C may also assist the SEC1 translocation pathway in translocating thylakoid precursor proteins from the stroma to the lumen (Jiang et al., 2020). Surprisingly, a SEC2 translocase system also exists that is similar to SEC1, but SCY2 and SECE2 are inner envelope membrane protein channels using the stromal motor protein SECA2 (Skalitzky et al., 2011). The known examples of SEC2-dependent translocation of inner envelope proteins are TIC40 and FTSH12 (Li et al., 2017). However, the SEC2 translocase system is poorly understood compared to SEC1 due to a lack of studies.

### TAT translocation mechanism

The Twin Arginine Transport (Tat) pathway is so called because the corresponding targeting sequences contain two neighbouring arginine residues (Cline et al., 1992). The TAT pathway is distinct from others in that it is able to transport fully folded protein across the thylakoid membrane and into the lumen. The TAT-specific signal sequence features are similar to those of SEC with the exception of the N-terminal part that contains the twin arginine (RR) motif. The RR motif is responsible for SEC avoidance response in thylakoid targeting (New et al., 2018). The Tat pathway is estimated to be responsible for the import of an estimated 50% of the thylakoid lumen proteins (Robinson and Bolhuis, 2004). The characteristic twin-arginine motif is essential for translocation and is disabled by mutation to other combinations of amino acids. The TAT pathway requires only the proton motive force (pmf) as energy source in order to achieve protein transport and has therefore also been called the ∆pH pathway (Mould and Robinson, 1991). Three proteins named TatC, Hcf106 and Tha4 form a complex that binds to the precursor protein’s RRXFLK motif in the N-terminal part of the signal sequence in order to facilitate translocation (Figure 1). Liquid-liquid phase separation by Hcf106-ankyrin-repeat proteins (STT) interaction facilitates the TAT dependent translocation of the luminal proteins (Ouyang et al., 2020). Several models of translocation have been proposed for the plant TAT pathway. However, no proven model exists to date (New et al., 2018).

### SRP

The chloroplast signal recognition particle (cpSRP) pathway, which is derived from prokaryotes and known as cpSRP pathway, targets and inserts abundant thylakoid membrane proteins, for example, light-harvesting chlorophyll-binding proteins (LHCPs) (Ziehe et al., 2018). Unlike SEC and TAT pathways, no conserved motif or domain is present at the N-terminal of the protein for thylakoid targeting. Several studies address LHCP recognition by the cpSRP pathway. The L18 motif (18 amino acids within the second and third transmembrane helices) of LHCP is crucial for recognition by cpSRP transit complex (Tu et al., 2000). Once nuclear-encoded LHCP is imported into the chloroplast via the TOC-TIC complex and processed by SPP, it forms the stromal transit complex together with cpSRP54 (GTPase) and cpSRP43 (Schuenemann et al., 1998). The cpSRP transit complex containing LHCP binds to cpSRP receptor cpFtsY (GTPase) (Kogata et al., 1999; Tu et al., 1999; Nguyen et al., 2011) and docks to Alb3 (insertase at thylakoid membrane) via cpSRP43, promoting precursor/LHCP insertion into thylakoid membrane (Figure 1) (Moore et al., 2000; Bals et al., 2010). cpSRP43 has two distinct chaperone activities for i) LHCP insertion and ii) tetrapyrrole biosynthesis enzymes. The chaperone activity towards tetrapyrolle biosynthesis activity allows to coordinate LHCP insertion with chlorophyll biosynthesis and assembly into LHCP. Interestingly, cpSRP54 activates the cpSRP43 chaperone function towards LHCP insertion and inhibits the chaperone activity towards tetrapyrrole biosynthesis enzymes (Wang et al., 2018; Ji et al., 2021). However, except for LHCP, there is a lack of information about how the SRP targets are recognized by the components of the SRP pathway.

### Thylakoid processing peptidase (TPP)

The thylakoid proteins are translocated into the thylakoid lumen by either the Sec or Tat pathways and, in a final step the N-terminal thylakoid targeting sequence is cleaved by Thylakoid Processing Peptidase (TPP) (Figure 1) (Hsu et al., 2011). TPP is a member of the membrane-bound proteases belonging to the type I signal peptidase (SPase I) family in both prokaryotes and eukaryotes. Plsp1 and Plsp2A/B are the two TPPs present in the thylakoids (Hsu et al., 2011). Plsp1 is known to be involved in the SEC and TAT dependent signal sequence cleavage and, surprisingly also, processing of TOC75 at the envelope membrane, suggesting that at least the Plsp1 protease is found not only in the thylakoid membrane but also in the envelopes. Plsp1 is essential for chloroplast biogenesis, its mutation resulting in a very pale green phenotype (Shipman and Inoue, 2009). Currently, the physiological and functional roles of Plsp2A/B in signal peptide processing are unclear.

## Conclusion and future perspectives

In the last years, significant progress has been made with regard to the understanding of the molecular and mechanistic details of chloroplast import of nuclear-encoded proteins by the TOC-TIC complex. The recent cryo-EM structural studies reveal how the TOC-TIC components are arranged in detail and provide some information on the likely path of the preprotein and its transit peptide across the chloroplast envelope. It would now be highly interesting to study the cryo-EM structure of the TOC-TIC complex in association with a preprotein and its transit peptide to gain a complete understanding of the import process. Also, the recent cryo-EM structures failed to reveal the cytosolic GTPase domains of the TOC34 and −159 (Jin et al., 2022; Liu et al., 2023) that play a central role in transit peptide recognition. The GTPase domains should remain a key target in future structural work. Recent advances in the chloroplast transit peptide field reveal that specific motifs, i.e., the proline-rich motif, have vital roles in the preprotein interaction with the TOC-TIC translocon. However, fundamental knowledge concerning the recognition and distinction of transit peptides belonging to different classes of preproteins (i.e., photosynthesis-associated vs. nonphotosynthetic housekeeping) is still lacking. In the future, the identification and investigation of particular motifs playing essential roles in tissue- and plastid-specific protein import pathways are predicted to be important research questions. Last but not least, many questions regarding second targeting sequences and their role in processing and assembly of the all-important photosystems remain open and should be addressed in the future.
